# Virtual Reality-Assisted Informed Consent for Anesthesia: A Prospective and Randomized Proof-of-Concept Study

**DOI:** 10.3390/jcm13206096

**Published:** 2024-10-12

**Authors:** Sebastian Simon, Philipp Opfermann, Jochen G. Hofstaetter, Peter Marhofer

**Affiliations:** 1 Department of Orthopedic Surgery, Orthopedic Hospital Speising, 1130 Vienna, Austria; sebastian.simon@oss.at (S.S.); jochen.hofstaetter@oss.at (J.G.H.); 2 Department of Anesthesia, Intensive Care Medicine and Pain Medicine, Medical University of Vienna, 1090 Vienna, Austria; philipp.opfermann@meduniwien.ac.at

**Keywords:** informed consent, orthopedic procedures, physician–patient relations, understandable communication, virtual reality, work-time efficiency

## Abstract

**Background/Objectives**: Informed consent for anesthesia poses both legal challenges and problems of understandable communication. Fulfilling all the requirements through anesthesiologists directly interacting with patients is a time- and staff-consuming strategy. Given today’s smart technologies, notably including virtual reality (VR), we explored in a prospective randomized study whether ‘VR-assisted informed consent’ could improve this situation. **Methods**: Fifty patients scheduled for orthopedic surgery were randomized. In the control group, informed consent was obtained via patient–specialist dialogs only. The patients in the study group, wearing a head-mounted display, watched an 8 min immersive 3D movie with the standard explanations of general anesthesia, followed by a patient–specialist dialog to address open questions. The time spent on the dialogs in both groups was evaluated as the primary outcome variable. As secondary variables, we analyzed both a three-item Likert scale on patient satisfaction with the VR experience and cost differences between both groups. **Results**: Patient–specialist dialogs were carried on for median (IQR) durations of 93 (20–182) seconds in the study group versus 665 (261–829) seconds in the control group (*p* < 0.001). All the patients exposed to VR rated this experience as favorable (87.5%) or neutral (12.5%). Based on anesthesiologists’ incomes in the US and UK, our approach would reduce the staff expenditure for each patient–specialist dialog by median amounts of USD ≈40 or ≈11, respectively (2 × *p* < 0.001). **Conclusions**: ‘VR-assisted informed consent’ for anesthesia is well accepted by patients and reduces the time requirements for patient–specialist dialogs, thus pointing out a potential avenue towards increasing the work-time efficiency of anesthesiologists.

## 1. Introduction

Informed consent is fundamental to perioperative patient management in general. With regard to obtaining such consent for anesthesia in particular, major challenges in daily clinical practice arise from legal requirements and from the need to explain procedures in a way that patients can actually understand. Inadequate information, either by too short an interval between consenting and the procedure or by content that is not readily comprehended, may engender uncertainty in patients and can even result in lawsuits [1]. Informed consent also needs to involve correct and reproducible documentation [2].

This challenging ethical and legal background prompted the publication, in 2017, of guidelines for consent by the Association of Anaesthetists of Great Britain and Ireland [3]. The American Society of Anesthesiologists, in turn, clearly states in its ethical guidelines that the “patient-physician relationship involves special obligations for the physician that include personal interaction with the patient, placing the patient’s interests foremost, faithfully caring for the patient and being truthful” [4]. The same source highlights the importance of adequately interacting with patients, requiring that anesthesiologists “respect the right of every patient to self-determination”.

There is an ever-present need to apply the highest ethical standards in daily clinical practice. Given the reports from around the world that patients do not fully grasp the messages communicated for informed consent [5,6,7], our quest to maintain these standards must include an effort to convey information as lucidly as possible to raise as many patients as possible to adequate levels of understanding. Various obstacles come into play here, including physicians’ everyday use of jargon that is not readily intelligible to patients, but factors such as age, cultural background, or level of education may also influence a given patient’s ability to adequately comprehend the information provided.

It is a fact that despite being a time- and staff-consuming medium, paper continues to be the gold standard of informed consent media in anesthesia [8]. Recent studies have explored alternatives like videos to illustrate urological procedures or ‘digital media’ for bariatric surgery [9,10], and preliminary reports are also available on virtual reality (VR) supporting the informed consent process for surgical procedures [11,12]. The present report follows suit by comparing ‘VR-assisted informed consent’ for anesthesia with the conventional ‘dialog-only’ method in terms of time requirements, patient satisfaction, and cost implications.

## 2. Materials and Methods

### 2.1. Trial Authorization

The protocol for this trial had been approved by the Vinzenz Gruppe institutional ethics committee (ref. 17/2022) on 7 July 2022, and the study had been registered with the German Clinical Trials Register (DRKS00029225) on 17 August 2022.

### 2.2. Study Design and Patients

Fifty adults who had been scheduled for elective knee or hip arthroplasty in a tertiary center (Orthopedic Hospital Speising, Vienna, Austria) were prospectively enrolled and randomized. A study group and control group were thus formed, where informed consent for anesthesia was obtained using either a VR-assisted approach or by relying entirely on the usual patient–specialist dialogs. An adequate command of German was the only inclusion criterion, and any patients with known epilepsy, vestibular disease, or vision disorder were excluded. All the participants were duly informed of the research objectives, and written informed consent for the study itself was obtained before the actual informed consent steps under investigation. The ‘VR-assisted’ procedures in the study group, including the dialogs to address open questions, were then followed by a regular process of informed consent to meet all the legal requirements for anesthesia.

### 2.3. Randomization and Control Group

A person not otherwise involved in the study used a web-based tool “www.randomizer.org (accessed on 20 August 2022)” for random assignment to a control or a study group. The randomization numbers thus returned were then used as identifiers for sealed envelopes containing the allocation. Two sets (one being a spare set for backup) of sealed envelopes that contained the allocation details were kept in a safe place throughout the study. One envelope was opened for each consecutive patient. The patients allocated to the control group were asked to complete a paper-based form for informed consent, approved by the Austrian Society of Anesthesiology, Resuscitation, and Intensive Care Medicine. This was followed by a one-on-one conversation with an anesthesiologist. All the specialists involved in patient dialogs during the study were blinded to the group assignment.

### 2.4. Study Group (‘VR-Assisted Informed Consent’)

**Figure 1:**
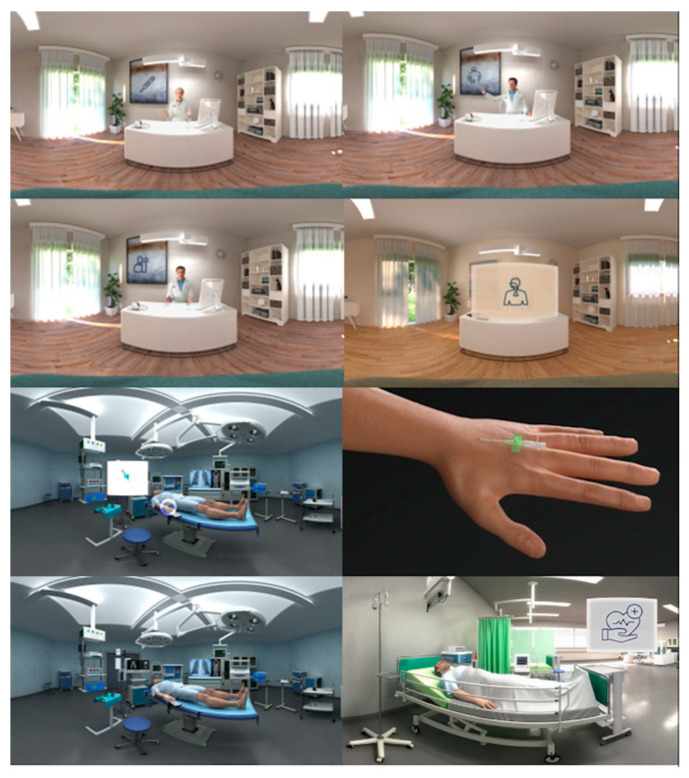
Individual frames from the 8 min VR movie.

The VR hardware consisted of a standalone head-mounted display (Pico Neo3 Pro/Pro Eye; Pico Interactive Europe, Barcelona, Spain) with preinstalled software that included a VR movie (XRSynergies; Vienna, Austria). This film, close to 8 min long, featured 3D rooms (clinician’s office, operating room) with doctors’ avatars giving routine information about general anesthesia and its risks. This content (individual frames are shown for illustration in Figure 1) had been developed from evidence-based guidelines issued by the German Network of Evidence-Based Medicine and the Austrian Society of Anaesthesiology, Resuscitation, and Intensive Care Medicine. A translation of the German language voice track is provided as Supplementary Text S1. For study purposes, the practical use of the head-mounted display was explained (S.S.).

A note was taken of any adverse events related to patients wearing the head-mounted display. Having watched the VR movie, each patient was asked to rate on a Likert scale, with applicable scores ranging from 1 (strongly agree) to 5 (strongly disagree), whether he or she felt that the audiovisual explanations had been a satisfying experience:

- Item 1: I experienced this VR-assisted way of giving my informed consent as useful.
- Item 2: I would use this VR-assisted way of giving my informed consent again.
- Item 3: I would recommend this VR-assisted method to my family and close friends.

### 2.5. Primary and Secondary Endpoints

The procedures in the study group just outlined were followed by one-on-one sessions with anesthesiologists for the patients to ask any open questions. The durations of the two different modes of patient–specialist dialog in both groups were recorded and analyzed as the primary outcome variable. As secondary variables, we analyzed the Likert scale items above and calculated, in US dollars, the cost differences between both groups in anesthesiologists’ time based on 1700 man-hours per year and average incomes earned by US [13] and UK [14] anesthesiologists in 2022.

### 2.6. Sample Size Calculation

We searched PubMed and Google Scholar for the effect sizes of VR interventions in studies dealing with informed consent. This returned one study analyzing comprehension and anxiety levels after informed consent procedures carried out either conventionally or using 3D VR [12]. Based on a remarkably high effect size for comprehension levels between both groups, we calculated post hoc a Cohen’s d of 2.48 and an effect size of r = 0.77 for the present study.

Our in-house records indicate a mean time requirement of ≈10 min for these patient–specialist dialogs. From the above literature search and considerations, we assumed a moderate-to-high effect size of Cohen’s d (>0.5) for this endpoint. A power analysis yielded a sample size of 50 patients for an 80% chance of detecting, at the 5% significance level, and a decrease from ≈600 ± 200 s (i.e., the 10 min just mentioned) in the control group to 435 s in the study group, allowing for a dropout rate of 8%. The intention-to-treat principle was applied to account for non-compliance, protocol deviations, withdrawal, or, indeed, any unforeseen events after randomization [15].

### 2.7. Statistical Analysis

A Kolmogorov–Smirnov test was used to check for normal distribution, followed by a non-parametric Mann–Whitney U-test for intergroup comparisons of metric and not normally distributed data. For intergroup comparisons of proportions, we used cross-tabulation and Pearson’s chi-square test. The results are expressed as medians with interquartile ranges (IQRs) and/or absolute values with percentages and differences considered significant at *p* < 0.05. All the operations were performed with the IBM^®^ SPSS^®^ statistical software (v. 26.0.0.0; IBM, Armonk, NY, USA) and other dedicated tools for data analysis (Prism v. 10.1.1; GraphPad Software, Boston, MA, USA).

## 3. Results

**Figure 2:**
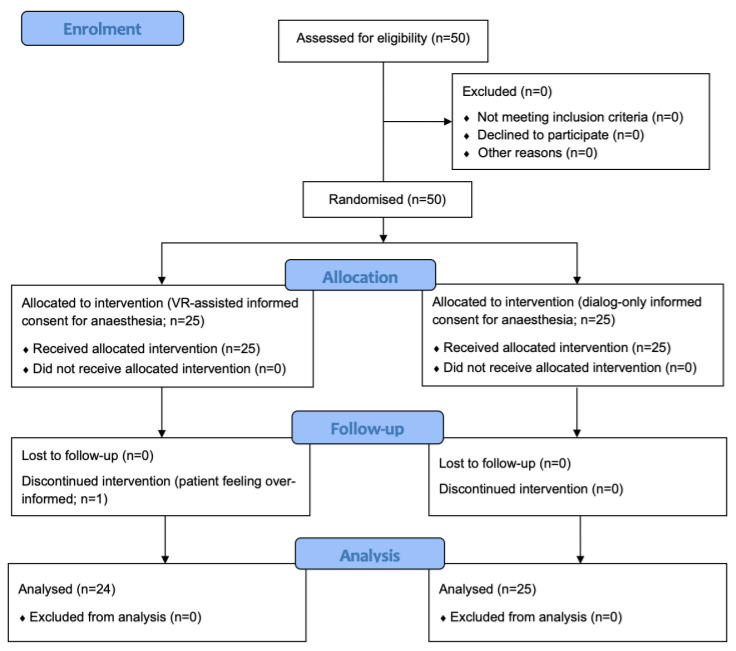
CONSORT flow diagram of the study.

Patient recruitment took place from 23 August 2022 to 6 April 2023. As required by the result of the sample size calculation, 50 patients were initially enrolled, 49 of whom completed the study. Figure 2 displays a flow chart of the study in accordance with the CONSORT guidelines. Pertinent patient data are summarized in Table 1. No adverse events were noted in connection with wearing the head-mounted display. We also did not detect any technical failures of the head-mounted displays or the preinstalled software.

In the study group, the patient–specialist dialogs preceded by exposure to the VR movie, which was itself 472 s (7:52 min) long, required a median of 93 s (IQR: 20–182 s). In the control group, the median duration of the conventional dialogs not preceded by exposure to a VR movie was 665 s (IQR: 260.5–829 s). This difference in median time requirements was statistically significant (*p* ≤ 0.001).

Table 2 lists the patient ratings in the study group for the three-item Likert scale. Note that all the patients exposed to the VR movie rated this experience as favorable (87.5%) or as neutral at worst (12.5%), while none of them indicated a negative impression. In addition, a vast majority (87.5%) affirmed both that they would use this VR-assisted method again and that they would recommend it to their family and close friends.

Table 3 illustrates the potential cost savings from the shorter dialogs in the study group. Based on average anesthesiologists’ incomes in the United States and United Kingdom, our method of ‘VR-assisted informed consent’ would reduce the cost of each informed consent procedure by around USD 40 or 10.7, respectively (*p* ≤ 0.001).

Cost differences in anesthesiologists’ time for each informed consent handled in the VR-assisted versus conventional way based on 1700 man-hours per year (6120.000 s) and average incomes earned by US [13] and UK [14] anesthesiologists in 2022. The values are expressed as US dollars.

## 4. Discussion

In this study, we compared a VR-assisted procedure of informed consent for anesthesia with the customary approach of placing the entire burden of information on patient–specialist dialogs. These were found to be significantly shorter with those patients who had previously watched the VR movie, thus reducing expenditures for anesthesiologists’ time. Also, the VR experience was found to be well accepted by the patients.

Informed consent is a mandatory requirement with both medical and legal implications. Despite an abundance of man-hours going into the conventional procedures of informed consent on a daily basis [8], there are limitations to how well these explanations of medical treatment are accepted and understood by the patients [5,7,16]. In neurosurgery, to name but one high-risk discipline, deficiencies in informed consent are central to about 10 percent of the malpractice claims [1].

Intelligibility to patients and the cost factor are two major concerns in connection with obtaining informed consent before medical treatment. As shown by Bai and colleagues [17], patients were scarcely able to recall the risks of interscalene blockade right after having been informed of them during the conversations. This is just one example of the dilemma existing between medico-legal eventualities by too little, and nocebo-style eventualities by too much, information. The effects of the latter type may arise from negatively biased expectations in patients comprehensively informed of all the potential risks and complications [18]. For truly informed consent, therefore, an adequate balance has to be struck that avoids both extremes.

Regarding the cost factor of informed consent procedures, Kieninger and colleagues [8] found 33 ± 16 min to be the mean time spent on patient–specialist dialogs for anesthesia. Also, experienced anesthesiologists were found to require significantly less time for patient interaction and documentation tasks. This may appear to suggest a greater involvement of experienced anesthesiologists in preoperative dialogs, but this goal could only be achieved at the expense of availability in operating rooms when the waiting lists for any kind of surgery are constantly growing.

As an alternative, it may reasonably be expected that the current generation of ‘smart’ technologies may increase the time and cost efficiency of informed consent procedures for anesthesia. ‘Digital media’ for bariatric surgery have been found to improve patients’ understanding of procedure-specific risks/benefits and to bring the time requirements from the conversation-only approach down by 50 percent [10].

VR, being the latest advancement in this area, offers 3D experiences that are much more emotionally stimulating than 2D media. Tian and colleagues [19] used multichannel electroencephalography and skin conductance responses, showing that more brain activity was evoked by 3D VR than by conventional 2D media. We are only beginning to understand the neuronal mechanisms underlying this experience. Further studies will be needed to corroborate the idea of 3D VR content performing significantly better than regular 2D visual media for informed consent.

Our study reveals that ‘VR-assisted informed consent’ for anesthesia can meet with high acceptance by patients. Even though our specific evaluation of patient satisfaction was confined to the study group, previous studies have demonstrated low degrees of overall satisfaction with the conventional patient–physician interactions used for preoperative informed consent [20]. Likewise, the degree to which anesthesia-related content thus communicated is actually understood by the patients has been questioned [21]. An instrument to assess the ‘understandability’ of audiovisual materials, a category broadly applying to our study group, is available in the form of PEMAT-A/V [22]. This is a tool, however, whose items were written to be answered by experts rather than by patients.

Also, for patients to watch an immersive VR movie covering all the relevant aspects of general anesthesia does not impose any additional requirements on medical staff. The approach we used for the study reduced the patient–specialist dialogs by two-thirds of the time spent on the conventional conversations. These data hold a promise, based on documented incomes earned by anesthesiologists in the US and UK, of cost savings on the order of USD 40.0 or 10.7, respectively, for each case handled in this way. It is relevant to mention that we did not include the costs for VR and software equipment in our calculations. Medical equipment is usually charged off for seven years and therefore these costs are neglectable when the high rate of applications and the costs for head-mounted displays are considered. In addition, we did not include the working time (and associated costs) of the initial explanation of the practical use of the head-mounted device. This explanation was performed by a medical doctor only for study purposes. In the later daily clinical practice, this explanation (if requested) can be performed by nursing staff.

The documentation tasks related to informed consent, too, are time-consuming activities with medico-legal implications. Negash and colleagues [7] found the documentation of informed consent to be inadequate in clinical practice. Even though speculative at this point, one consideration would be to explore the possibilities of eye tracking offered by head-mounted displays (like the device used in the present study) as a means to improve documentation. On an even more speculative note, and with legal requirements allowing, one might toy with the idea of eye tracking one day superseding the need for handwritten signatures [23].

Language barriers and the related problems of accessibility pose yet another challenge to informed consent. While the present study was confined to German language material for ‘VR-assisted informed consent,’ obviously its content can be personalized in various ways. Not only could the material be produced in any useful languages (reducing the need and outlays for interpreters to any questions the patients may still want to ask the anesthesiologist after having watched the movie), but its content could also be fine-tuned to reflect sociocultural differences, thereby improving comprehension of consent-relevant information by patients belonging to specific population groups.

As a related thought, readily understandable formats of preoperative information also gain importance as increasingly older patients are undergoing surgery. Giampieri and colleagues [5] identified three important considerations in this regard: (i) individual cognitive function; (ii) degrees of cognitive impairment; and (iii) legal guardianship. Theoretically, it should be possible for artificial intelligence [24] and VR to adapt their conveying of information to different degrees of cognitive impairment. That being said, while more studies are needed to shed light on VR-assisted informed consent, it seems clear enough that older patients as such are perfectly able to use and understand VR [25].

Limitations of the present study include, first of all, that patient satisfaction with the VR experience was tested in the study group (‘VR-assisted informed consent’) but not in the control group (conventional dialogs only) and that a tool similar to PEMAT-A/V (designed for use by experts) to assess the ‘understandability’ of audiovisual materials from the patients’ perspective was not available. We do, however, know from previous studies that many patients are dissatisfied with the informed consent process and that their grasp of the information thus provided is limited [20]. Second, our VR movie did not cover regional anesthesia, but note that this study was only a first step in evaluating VR for informed consent in anesthesia and that subsequent versions of the movie will cover both general and techniques of regional anesthesia.

In summary, a sample of patients scheduled for orthopedic surgery was prospectively randomized to compare a VR-assisted method with the traditional dialog-only method of obtaining informed consent for anesthesia. This study revealed that ‘VR-assisted informed consent’ was well accepted by the patients without involving any observable side effects or complications. We demonstrated a significant potential for cost savings with the VR-assisted method due to reduced time requirements for anesthesiologists engaging in patient dialogs. Well-designed clinical studies are needed to confirm these results of ‘VR-assisted informed consent’ for anesthesia in other fields of surgery.

## Figures and Tables

**Table 1:** Pertinent patient data. Values are expressed as medians with interquartile ranges (IQR) or as absolute numbers (n).

	VR-Assisted (Study Group)	Dialog Only (Control Group)	*p*-Value
Age	58 (42–67)	63 (55–69)	0.28
Biological sex (m/f)	13/11	15/10	0.77
BMI (kg m^−2^)	26 (24–29)	29 (28–32)	0.04
ASA classification (1/2/3)	13/11/0	10/13/2	0.28

**Table 2:** Patient ratings of their VR experience on a three-item Likert scale. Values are expressed as absolute numbers (n) and percentages (%).

	Strongly Agree	Agree	Neutral	Disagree	Strongly Disagree
I experienced this VR-assisted way of giving my informed consent as useful.	19 (79.2)	2 (8.3)	3 (12.5)	–	–
I would use this VR-assisted way of giving my informed consent again.	19 (79.2)	2 (8.3)	1 (4.2)	1 (4.2)	1 (4.2)
I would recommend this VR-assisted method to my family and close friends.	19 (79.2)	2 (8.3)	1 (4.2)	1 (4.2)	1 (4.2)

**Table 3:** Cost differences in anesthesiologists’ time for each informed consent handled in the VR-assisted versus conventional way based on 1700 man-hours per year (6120.000 s) and average incomes earned by US [13] and UK [14] anesthesiologists in 2022. The values are expressed as US dollars.

	Annual Income	Hourly Income	Income per Second	VR Assisted (Study Group)	Dialog Only (Control Group)	Δ	*p*-Value
US	432,500.00	254.12	0.07	6.57	46.55	39.98	<0.001
UK	114,712.00	67.48	0.02	1.74	12.46	10.72	<0.001

## Data Availability

Anonymized data will be made available upon reasonable request. Please contact the corresponding author.

## Supplementary Materials

The following supporting information can be downloaded at https://www.mdpi.com/article/10.3390/jcm13206096/s1, Supplementary Text S1: A translation of the German language voice track.
