# Supplementary material for: Virtual Reality-Assisted Informed Consent for Anesthesia: A Prospective and Randomized Proof-of-Concept Study
Source: J Clin Med. 2024 Oct 12;13(20):6096. doi: 10.3390/jcm13206096 (PMC11509058; doi:10.3390/jcm13206096)
Supplement: Supplementary file 1 [file jcm-13-06096-s001.zip › jcm-3250090-supplementary.pdf]

Script for virtual reality-based educational video

GENERAL ANESTHESIA

Procedures and potential risks

Good day, I am your Me-Doc!

My task is to thoroughly inform you about the procedure and the risks of general anesthesia.

What is important before general anesthesia?

- Adults should not eat 6 hours and should not drink 2 hours before the operation.
- Please avoid smoking for as long as possible before and after your operation.
- Please inform your treating doctor if you have a cold or feel unwell.

What is general anesthesia?

Anesthesia can be compared to deep sleep. There is also the possibility of half-sleep (also known as sedation), which is used in combination with local anesthesia. In half-sleep, you are given sedation medication, but you can still be woken up and will breathe on your own.

In both cases, your anesthesiologist will always be there to monitor and care for you. However, it is important to note that no medical procedure is without risk, and despite utmost care, complications may occur in some cases. These are generally rare, and it is important to know that life-threatening events or cases of permanent damage are exceptionally rare.

How does general anesthesia work?

Before the operation, an IV line will be placed, usually using a vein in the hand or forearm. Sometimes, bruising or inflammation may occur at the insertion site.

If the situation requires, your doctor may insert a "central venous catheter" into one of the large veins. When this is done in the upper body area, lung injury may occur as a very rare complication.

©XRSynergies LLC 2022 - Lagergasse 6/10 - 1030 Vienna - www.xrs-medical.com

All medications you receive before, during, and after anesthesia can occasionally cause allergic reactions, such as itching, problems with breathing, or circulatory problems. The same applies to materials like adhesive plasters, as well as to local anesthetics. Another very rare complication of anesthesia medications would be a sudden onset of high fever.

Let me briefly return to the fasting rule mentioned at the beginning because, as you are falling asleep, there is a risk that your stomach might empty, and stomach contents could enter the lungs, potentially leading to severe lung damage. If your stomach is empty, this risk is significantly lower.

Once you are deeply asleep, your doctor will securely take over your breathing with a breathing tube or mask.

It is very rare for teeth to be damaged during this process. Please be sure to inform your doctor if you currently have loose teeth or wear a removable denture.

As a result of the breathing tube, you might experience mild throat pain or an odd sensation when swallowing after waking up. This usually only lasts for a short time.

What other complications might occur?

- Formation of blood clots in the body, which can lead to organ damage. The likelihood of this happening depends heavily on the kind and duration of the operation, and you may be given medication to reduce this risk.
- Temporary heart, circulatory, or respiratory issues.
- Nerve or tissue damage due to your lying position during the operation; in rare cases, eye injuries might occur.
- In some cases, the half-sleep I have explained before may need to be converted to full-blown general anesthesia, meaning that all anesthesia risks are also relevant to half-sleep.
- Gaining consciousness or waking up is extremely rare during anesthesia, occurring in only 0.01−0.2% of the cases.

Additional Measures

Additional measures may become necessary in relation to anesthesia, including:

- Insertion of a urinary catheter, which may cause minor injuries or urinary tract infections.
- Placement of an arterial line, usually at the wrist, with possible complications like infection, bruising, or nerve damage.
- Temporary insertion of a gastric tube, carrying a risk of injury to the stomach or lungs.
- Transfusion of blood, which today is very safe, with rejection reactions or the transmission of infections being extremely rare.

Whether these measures are necessary for you will be decided by your attending doctor.

What happens after the operation?

You will be taken to the recovery room and further monitored. You may feel confused for a while after anesthesia. You may also experience nausea, vomiting, or shivering, but this can usually be fixed easily and quickly.

Please discuss any questions you may have with your treating doctor.

I wish you all the best!
